# Hospital employees' theoretical knowledge on what to do in an in-hospital cardiac arrest

**DOI:** 10.1186/1757-7241-18-43

**Published:** 2010-08-09

**Authors:** Marie-Louise Södersved Källestedt, Andreas Rosenblad, Jerzy Leppert, Johan Herlitz, Mats Enlund

**Affiliations:** 1 Uppsala University, Centre for Clinical Research, Central Hospital, Västerås, Sweden; 2 University of Gothenburg, Sahlgrenska University Hospital, Gothenburg, Sweden

## Abstract

**Background:**

Guidelines recommend that all health care professionals should be able to perform cardiopulmonary resuscitation (CPR), including the use of an automated external defibrillator. Theoretical knowledge of CPR is then necessary.

The aim of this study was to investigate how much theoretical knowledge in CPR would increase among all categories of health care professionals lacking training in CPR, in an intervention hospital, after a systematic standardised training. Their results were compared with the staff at a control hospital with an ongoing annual CPR training programme.

**Methods:**

Health care professionals at two hospitals, with a total of 3144 employees, answered a multiple-choice questionnaire before and after training in CPR. Bootstrapped chi-square tests and Fisher's exact test were used for the statistical analyses.

**Results:**

In the intervention hospital, physicians had the highest knowledge pre-test, but other health care professionals including nurses and assistant nurses reached a relatively high level post-test. Improvement was inversely related to the level of previous knowledge and was thus most marked among other health care professionals and least marked among physicians.

The staff at the control hospital had a significantly higher level of knowledge pre-test than the intervention hospital, whereas the opposite was found post-test.

**Conclusions:**

Overall theoretical knowledge increased after systematic standardised training in CPR. The increase was more pronounced for those without previous training and for those staff categories with the least medical education.

## Introduction

The survival rate after cardiac arrest depends on the quality of cardiopulmonary resuscitation (CPR), alarm response time, and time to defibrillation [1,2]. All health care professionals should be able to perform CPR with competence [3]. Studies have investigated and compared different ways of teaching CPR with the aim to find a gold standard, maximising the best retention of knowledge [4-6]. Some studies have also suggested that too much emphasis is placed upon verbal information and too little on practical skills during training [5,7,8].

In the year 2000, CPR guidelines recommended that health care professionals should use an AED as soon as possible during CPR [9]. In order to be able to perform CPR effectively, however, they must first possess a theoretical knowledge of the subject. Previous studies have mostly investigated the CPR knowledge of nurses [10,11]. One of these studies, for example, stated that accurate knowledge of CPR guidelines was associated with a better chest compression rate and compression to ventilation ratio [10]. In another study including a mixed group of 224 medical students and physicians, an improvement in CPR knowledge was recognised after training [12]. Furthermore, in another study investigating healthcare professionals at a hospital, CPR skills nine months after education were self rated to be 3.8 in a five point scale with 1 = very bad and 5 = very good [13].

It remains the case, though, that some hospitals in Sweden, including one in the authors' county, lack the organisation for repeated CPR education and training (personal communication with S. Aune, Swedish Resuscitation Council, December, 2009). In the current study, a majority of health care professionals at two hospitals were available to investigate the impact of adult CPR training on CPR theoretical knowledge. At one of the two participating hospitals all the staff, except for those in two specialised units, were devoid of CPR training and education for several years due to reorganisation. The aim was to investigate how much theoretical knowledge in CPR would increase among all categories of health care professionals after a systematic standardised training.

The hypothesis was that theoretical knowledge would increase in all groups of health care professionals, and that the intervention hospital would reach the level of the control hospital. Secondary objectives were to assess if increase in theoretical knowledge was directly related to the level of previous knowledge.

## Methods

The study was approved by the regional ethics committee (Dnr 2006/201). Health care professionals were recruited at one intervention- and one control hospital in the county of Västmanland, Sweden (a total of 3144 individuals).

### Intervention and control

The study started in early 2006 [14]. Data collection was completed during 2009, at which time all employees had received CPR education. The effect of an introduced education programme (= intervention) was measured by a questionnaire concerning theoretical knowledge in CPR. It was then compared with the level of knowledge before the intervention and with the level of knowledge at a second hospital with an ongoing annual CPR training programme. Before training, the intervention hospital had approximately 20 CPR instructors. As training in CPR had not been organised for several years, with the exceptions of ICU and coronary ward staff, 30 additional instructors were trained, as were five leading instructors. The aim was that every ward at the intervention hospital should have two instructors. The instructors were not aware of the questions. AEDs were obtained and installed at the intervention hospital on May 1, 2007. The established organisation for CPR at the control hospital followed Swedish national guidelines, and every ward at this hospital had an AED from the year 2003.

The study period included two different CPR guidelines, from 2001 and 2005. The pre-test questionnaire was evaluated according to the guidelines from 2001, still in use in early 2006, and the post-test questionnaire was evaluated according to those from 2005, implemented in late 2006. After the pre-test, the instructors were educated in the new guidelines. The training in CPR at both hospitals was standard instructor led CPR training, following the Swedish national education programme [15]. The intervention was a four hours fundamental course with a mixture of theory and practical training (basic life support + AED). The control hospital's employees received a repetition course in basic life support + AED, taking 2 1/2 hours according to the National education programme, focusing on news in guidelines and on practical training.

### Participants

**Figure 1:**
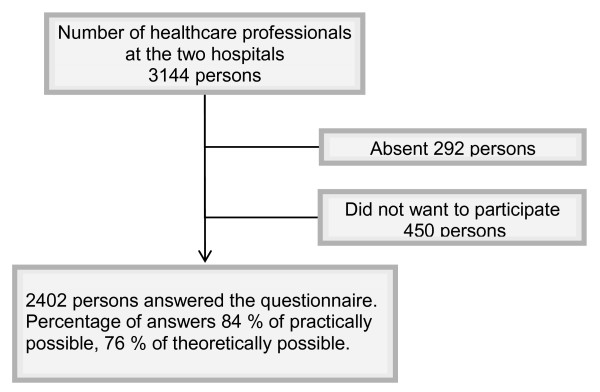
**Number of healthcare professionals invited and participating, pre-test**.

**Figure 2:**
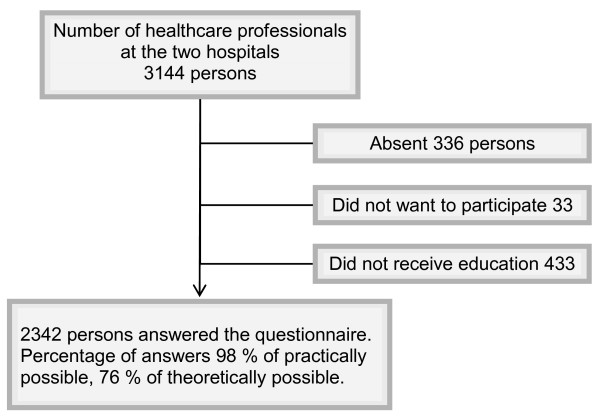
**Number of healthcare professionals invited and participating, post-test**.

The number of participants in the two parts of the study is presented in Figures 1 and 2. All healthcare professionals available at the two hospitals were invited to participate. Those eligible for inclusion in the study were actively working at the time, i.e., those on maternity- or sick leave was not included. To be eligible for the post-test it was also required that the individual had actually participated in training. The participants were divided according to their professions into the following five groups: physicians, nurses (including midwifes), other university educated staff (including physiotherapists, occupational therapists, social welfare officers, psychologists and biomedical analysts), assistant nurses (including keepers), and finally other remaining occupational groups such as secretaries, kitchen and service staff (when these groups were involved in active patient care). Table 1 presents the participants according to their professions.

**Table 1:** Distribution of 3144 health care professionals participating in the study, according to their medical profession.

Profession	Control hospital "**Before" (n)**	Control hospital "After" (n)	Intervention hospital "Before" (n)	Intervention hospital "After" (n)
Physicians	16 (6.2%)	8 (2.7%)	248 (12%)	204 (10.2%)
Nurses	100 (38.9%)	132 (43.9%)	905 (43.7%)	910 (45.4%)
Assistant nurses	90 (35%)	116 (38.5%)	645 (31.1%)	621 (31%)
Other university- educated staff	37 (14.4%)	36 (12%)	175 (8.4%)	120 (6%)
Others	14 (5.4%)	9 (3%)	100 (4.8%)	148 (7.4%)
Total with information about profession	**257**	**301**	**2073**	**2003**
No information about profession	6	7	65	31

**Total**	**263**	**308**	**2138**	**2034**

(n) = number

### Questionnaire

The authors developed and validated a multiple-choice questionnaire to investigate the health care professional's theoretical knowledge of CPR [16]. This questionnaire covers the following areas: evaluation of an unconscious patient, chest compressions, mouth-to-mouth ventilation, and defibrillation (cf. Appendix). It was developed from study questions obtained from the Swedish Society of Cardiology education programme [14,17] and contains 15 questions, all with only one correct answer. The questionnaire was to be completed 4-12 weeks before and 0-8 weeks after CPR training at the intervention hospital. The staff at the control hospital completed the questionnaire during the same period as their annual repeat training. The questionnaires were distributed on paper using the hospitals' internal mailing systems.

### Statistical analyses

In order to increase the response rate it was decided that the questionnaires should be answered anonymously, thereby eliminating any potential concerns among participants of the possibility of tracking individual results. With a staff turnover rate of 8.2 percent per year, it could be expected that about 85 percent of the health care professionals that answered the post-test questionnaire had also answered the pre-test questionnaire. This implies that the pre- and post-test answers were correlated. In the statistical analyses for comparing pre- and post-test results, this would usually be taken care of by pairing the pre- and post-test answers from the same person. However, since the questionnaires were answered anonymously, this was not possible. Thus, the pre- and post-test answers were correlated without being paired, meaning that the standard p-values from common statistical tests of significance such as Pearson's χ^2^-test, which requires independent variables, or McNemar's test, which requires paired variables, could not be trusted. Instead, one has to resort to bootstrapping [18] for calculating reliable p-values for this situation. After dichotomising the answers to each of the 15 questions in the questionnaire as either right or wrong, the bootstrapping procedure applied a χ^2^-test for two independent proportions to the pre- and post-test answers, using 10 000 bootstrap resample's, to get the bootstrapped p-values. The calculations were performed in the statistical software R [19] and used the standard non-parametric percentile method to calculate the p-values. To compare the results on the test cross-sectionally between the health care professionals at the two hospitals pre- and post-test, respectively, bootstrapping was not necessary, since the two hospitals were independent. For this analysis, Fisher's exact test was calculated both pre- and post-test using SPSS statistics 17.0 [20]. For all statistical tests, a two-sided p-value of < 0.05 was considered to be statistically significant. Since bootstrapped p-values have an inherent variability, a value of < 0.045 was considered statistically significant for these, to ensure that the bootstrapped p-values did not exceed the 0.05 level.

## Results

The mean age of the health care professionals working at the two hospitals was 46.8 years (range 18-74). The number taking part in the study at the control hospital was 308, with a mean working experience of 20.7 years (range from a few months to 44 years). The corresponding number of participants at the intervention hospital was 2034, having, on average, 17.8 years (range from a few months to 46 years) of working experience.

The participants completed the questionnaire for the second time 2-8 weeks after training. A small group from the intervention hospital (n = 140) completed the questionnaire immediately after their training. Their results did not differ from the others. The internal percentages of missing answers varied between 0.7-13.5 percent in the questionnaires.

### Main findings

Overall, the staff at the intervention hospital presented significantly better results post-test compared with pre-test. Comparing the two hospitals, the staff at the control hospital presented a significantly higher level of knowledge pre-test, whereas at the intervention hospital staff performed significantly better post-test (Table 2).

**Table 2:** Result as percentage of correct answers, pre and post- test, and p-values at the intervention- vs. the control hospital.

	Intervention	Control		Intervention	Control	
**Question**	**Pre%**	**Pre%**	***P-value***	**Post%**	**Post%**	***P-value***

1	87	86	0.564	94	94	0.899
2	16	15	0.858	46	27	<0.001
3	41	65	<0.001	86	71	<0.001
4	65	80	<0.001	80	84	0.090
5A	50	62	<0.001	69	62	0.040
5B	22	30	0.008	38	27	0.003
5C	44	51	0.039	63	58	0.166
5D	20	28	0.011	24	25	0.614
6	47	70	<0.001	89	77	<0.001
7	54	71	<0.001	87	82	0.019
8	33	50	<0.001	76	62	<0.001
9	4	8	0.010	26	15	<0.001
10	49	58	0.007	63	63	0.899
11	97	99	0.148	99	100	0.160
12	71	77	0.067	89	83	0.002
≥50%	39	62	<0.001	84	73	<0.001
≥80%	8	12	0.019	30	21	0.001
100%	0.0	0.8	0.301	0.0	0.6	0.157

≥50% = the percentage of participants having more than eight correct answers

≥80% = the percentage of participants having more than twelve correct answers

### Intervention hospital - findings from a staff category perspective

The group containing other university-educated staff increased their number of correct answers more than any other group from pre- to post-testing (Table 3). The two groups of nurses and assistant nurses increased their results significantly in the areas of evaluating an unconscious patient and defibrillation. Physicians presented the highest number of correct pre-test answers compared with all other groups, and they did not significantly increase this result. At post-test, nurses and physicians had equal results.

**Table 3:** Percentage of correct answers at the intervention hospital according to medical profession, pre- and post-test, and bootstrapped p-values.

	Physicians			Nurses			Assistant Nurses			Other university-educated staff			Other occupational groups			Total	Total	
**Q**	**Pre%**	**Post%**	***P-value***	**Pre%**	**Post%**	**P-value**	**Pre%**	**Post%**	***P-value***	**Pre%**	**Post%**	***P-value***	**Pre%**	**Post%**	***P-value***	**Pre%**	**Post%**	***P-value***

1	92	93	0.718	89	94	0.040	86	94	0.013	80	95	0.025	78	88	0.208	87	94	<0.001
2	18	39	0.015	19	49	<0.001	13	49	<0.001	10	45	<0.001	18	29	0.221	16	46	<0.001
3	64	87	0.005	44	87	<0.001	36	88	<0.001	22	91	<0.001	24	71	<0.001	41	86	<0.001
4	60	62	0.701	76	85	0.014	65	84	<0.001	40	72	0.009	35	60	0.056	65	80	<0.001
5A	97	92	0.138	59	76	<0.001	33	63	<0.001	18	44	0.021	16	26	0.246	50	69	<0.001
5B	50	61	0.193	27	43	0.001	11	32	<0.001	2	9	0.150	6	8	0.519	22	38	<0.001
5C	72	83	0.127	48	65	0.002	38	60	0.001	17	49	0.007	18	37	0.101	44	63	<0.001
5D	56	54	0.631	24	27	0.271	7	15	0.057	3	5	0.491	2	7	0.299	20	24	0.135
6	86	93	0.170	52	90	<0.001	38	92	<0.001	18	97	<0.001	15	65	<0.001	47	90	<0.001
7	68	81	0.099	61	90	<0.001	54	92	<0.001	20	89	<0.001	34	57	0.069	54	87	<0.001
8	64	79	0.082	36	79	<0.001	29	80	<0.001	7	76	<0,001	15	41	0.024	33	76	<0.001
9	4	13	0.071	4	29	<0.001	4	30	<0.001	0	15	<0.001	4	9	0.284	4	26	<0.001
10	46	58	0.155	53	66	0.009	47	62	0.014	41	58	0.120	42	58	0.174	49	63	<0.001
11	100	99	NA	99	99	0.765	97	99	0.037	90	100	0.001	92	96	0.342	97	99	0.020
12	76	88	0.088	77	91	<0.001	69	89	<0.001	52	86	0.001	60	84	0.038	71	89	<0.001
≥50%	86	90	0.306	45	88	<0.001	27	86	<0.001	10	80	<0.001	15	47	0.005	39	84	<0.001
≥80%	18	36	0.033	12	37	<0.001	3	26	<0.001	0	1	<0.001	2	7	0.204	8	30	<0.001
100%	0	0	NA	0.0088	3.4	0.037	0	0	NA	0	0	NA	0	0	NA	0.003	0.018	0.009

Q = Question

NA = Not Available

Pre% = Percentage points correct answers before training

Post% = Percentage points correct answers after training

≥50% = the percentage of participants having more than eight correct answers

≥80% = the percentage of participants having more than twelve correct answers

P-values less than 0.045 were considered significant.

### Intervention hospital - findings related to specific questions

To the question "How soon should you defibrillate?" a large number of health care professionals answered that it should be performed within one minute. According to Swedish guidelines the time frame is three minutes. The number of correct answers to the question regarding which kind of arrhythmia to defibrillate increased significantly for several groups, mostly for the group of assistant nurses. To the question "Where to place the defibrillator electrodes on the patient during CPR?" all health care professionals increased their knowledge except for physicians who already presented a good level of knowledge at pre-test. All health care professionals proved to do well in questions about ventilation at pre-test, and the results did not improve post-test.

## Discussion

### Main findings

Standardised training in CPR is expected to be associated with improvement in many aspects of resuscitation. In this article we address one of them: theoretical knowledge. Our main finding was that from a hospital perspective, standardised education in CPR was associated with improvement in theoretical knowledge in CPR. The staff category (Table 3) had effect on the knowledge before CPR education. This effect was reduced after education. To the best of our knowledge this information is new and therefore unique.

### Intervention hospital - findings from a staff category perspective

The strength of the current study is the large sample of different healthcare professional categories who participated, representative of the entire spectrum of staff in a relatively large hospital and one small hospital. Previous studies have mostly investigated nurses or candidates [10-12]. Additionally, all participants were investigated both before and after their education.

Theoretical knowledge about how to perform CPR is essential for the ability to perform it in practise. It has been previously illustrated that nurses with good theoretical knowledge achieve better CPR performance [10]. In another study, theoretical knowledge among nurses was shown to increase after training but their skills did not [21]. In a study concerning cardiologists, it was proven that this group had such good theoretical knowledge from start, that they did not substantially increase it after training [12]. This concurs with the results of the current study, in which physicians had good knowledge pre-test but had not improved it significantly post-test. In contrast to this, the groups of other university-educated staff and the assistant nurses, both starting from a low level, markedly increased their theoretical knowledge. All instructors had passed instructor training and they strictly adhered to the standard teaching programme. This was supported by the fact that the post-test results did not significantly change for physicians. Other studies, which included staff categories such as nurses and physicians, supported the finding that theoretical knowledge will increase after CPR training [10,22]. The current study adds that this increase in knowledge concern all different kinds of healthcare professionals, at least those who start from a low level of knowledge.

### Intervention hospital - findings related to specific questions

Our questionnaire included four questions regarding which arrhythmia to defibrillate. Skrifvars and colleagues [23] demonstrated that AEDs eliminate some of the problems in association with rhythm analysis. We agree with Skrifvars, that these questions are not relevant for CPR training, as the AED itself indicates when to defibrillate. As expected, the highest internal missing rate, 8-13.5 percent, was noted for these four questions. Only specialists are expected to have this knowledge. When excluding these questions, the internal percentages of missing answers varied between 0.7-2.3 percent. For convenience, these questions are grouped together as 5A-D in Table 3.

### Findings from a hospital perspective

Why did the staff at the intervention hospital perform better post-test compared with their colleagues at the control hospital? One explanation may be that the training effort at the intervention hospital was of an extraordinary nature, combined with the placement of AEDs around the hospital, which may have had the charm of novelty. In contrast, at the control hospital, AEDs had already been in place for several years and the staff followed a well-known ongoing training programme, which was 1 1/2 hour shorter than at the intervention hospital.

### General discussion

We wanted to capture all employees at the two hospitals. Then, we choose to separate physicians and nurses into different groups, following the designs in other studies [10,12]. Assistant nurses constituted another group, since they lack a university degree but they work very close with patient care. Other healthcare professionals with a university education, but without close patient care, formed a third group. Registered professionals are enjoined by law to update themselves on new items [24]. The last group, "other occupational groups", meets patients and has some patient care, although they do not take part in immediate patient care. With this grouping, all employees with any patient contact were grouped in the most functional way.

The groups of physicians and nurses had the highest numbers of participants with more than 80 percent correct answers post-test (36 and 37 percent fulfilled this criterion). It is appropriate that these groups of health care professionals have the best knowledge in CPR, being the two groups with the main responsibility for providing medical care. The important finding that physicians presented better knowledge pre-test than the other health care professionals, may indicate that they read and update themselves. Specifically, physicians did better in questions regarding arrhythmias, a difference that to some extent remained at post-test. Bearing in mind the distribution of responsibility during CPR, with or without the use of AED, such a difference between professional groups seems adequate. The group of other health care professionals increased their theoretical knowledge most of all groups, as they started from an inferior level of knowledge. One conclusion might then be that training may compensate for poor basic knowledge. Repeated education and training may further increase knowledge, or at least maintain it at a certain minimum level.

The decline in CPR knowledge and skills started as early as three months after the training of lay-people [25]. Another study showed that practice and frequent participation in CPR incidents have a positive effect on knowledge[26]. Our study did not test long-term retention of CPR knowledge.

### Limitations

It is expected that healthcare professionals should have theoretical knowledge of CPR. This may place some stress on a potential study participant. Consequently, we chose not to have any identification number for the participants. Anonymity resulted in a good response rate, although it did so with the need for a more complex statistical analysis. The advantage of using bootstrap in the analyses is that this method takes care of the dependency between the pre- and post-test results and produces reliable p-values. A disadvantage is that the number of bootstrap resamples has to be limited to be computationally feasible, and thus variation is introduced into the p-values. However, this disadvantage was eliminated as we chose a bootstrapped p-value of < 0.045 to be considered statistically significant, which is equivalent to a non-bootstrapped p-value of < 0.05.

New CPR guidelines were introduced shortly after the first questionnaire was completed (2005). Our interventional CPR training therefore followed the new 2005 guidelines, and the post questionnaire was evaluated according to these guidelines. The content of the questionnaire was constructed in such a way that the mixing of the two guidelines during the study period would not influence the results.

Since the questionnaire was distributed with internal mail in paper format, we did not know if some of the healthcare professionals received help from the guidelines or from each other while answering the questionnaire. However, a majority of the participants answered the questionnaire during supervised working time.

The results from the control hospital may be difficult to evaluate, as it was hard to maintain the 2-8 weeks time frame for follow-up. This was due to irregularity in the continuing programme. Thus, the results from the control hospital may be falsely inferior.

## Conclusion

The main finding of this study was that CPR theoretical knowledge increased with training at the intervention hospital. Here, the training was most effective in the group containing "other university-educated staff", the group that performed worst before training. Physicians, starting from a high educational level, did not improve significantly in contrast to nurses who presented results after training comparable with the physicians.

## List of abbreviations

AED: automated external defibrillator; CPR: cardiopulmonary resuscitation; ICU: intensive care unit.

## Competing interests

The authors declare that they have no competing interests.

## Authors' contributions

MLSK participated in the design and planning of the study, carried out the data collection, wrote the manuscript draft, and co-ordinated the following versions of the manuscript. MLSK also partly participated in the statistical analysis. ME participated in the design and planning of the study and were involved in drafting the manuscript to an intellectual content. Also, he partly participated in the statistical analysis. JL participated in the design and planning of the study and revised the manuscript. JH revised the study and made important intellectual additions. AR performed the statistical analysis and partly revised the manuscript. All authors read and approved the final manuscript.

## Appendix

Multiple-choice questionnaire, after each question correct answers are presented. The correct answers are according to the Swedish national guidelines.

1. What is the first thing you should do if you see a person collapse in the waiting room of the hospital where you work?

Correct answer: Check for response, breathing and pulse

2. How long a time (in seconds) should your inspection of a patient with suspected cardiac arrest take? 

Correct answer: 30 seconds

3. What first aid equipment should you prioritise if you are unable to obtain all the necessary first aid equipment immediately?

Correct answer: Defibrillator

4. Can health care professionals working at the hospital use an automatic external defibrillator?

Correct answer: Yes but only persons who has passed a CPR course with an AED

5 A-D. At which arrhythmia should you defibrillate during ongoing CPR?

Correct answer: Ventricular fibrillation and pulse less ventricular tachycardia

6. Where should you place the defibrillator electrodes on the patient during CPR?

Correct answer: One below right clavicle and the other 10 cm below left armpit

7. The patient is soaking wet with cold sweat, what should you do to be able to defibrillate?

Correct answer: Dry the area where the electrode plats should be placed and the area between the plates

8. How many times in one sequence can you defibrillate during ongoing CPR?

Correct answer: Maximum one defibrillation at the time, then you has to do CPR

9. The patient has ventricular fibrillation at the first rhythm section. How soon should you defibrillate according to the existing guidelines?

Correct answer: Within 3 minutes

10. In connection with CPR, what should you do when you give breaths or ventilate?

Correct answer: Breath/ventilate slowly

11. How do you know that the breaths or the ventilation are effective?

Correct answer: You see the chest rising

12. With which frequency (minutes) should you perform chest compressions?

Correct answer: 100 compressions/minute
